# The ability to recognize dog emotions depends on the cultural milieu in which we grow up

**DOI:** 10.1038/s41598-019-52938-4

**Published:** 2019-11-11

**Authors:** Federica Amici, James Waterman, Christina Maria Kellermann, Karimullah Karimullah, Juliane Bräuer

**Affiliations:** 1 Research Group “Primate Behavioural Ecology”, Department of Human Behavior, Ecology and Culture, Max Planck Institute for Evolutionary Anthropology, Leipzig, Germany; 2 Behavioral Ecology Research Group, Institute of Biology, Faculty of Life Science, University of Leipzig, Leipzig, Germany; 3 Leipzig Research Center for Early Child Development, University of Leipzig, Leipzig, Germany; 4 School of Psychology, University of Lincoln, Lincoln, UK; 5 Faculty of Social and Behavioral Sciences, Friedrich Schiller University, Jena, Germany; 6 Department of Linguistic and Cultural Evolution, Max Planck Institute for the Science of Human History, Jena, Germany; 7 Friedrich Schiller University, Department of General Psychology and Cognitive Neuroscience, Jena, Germany

**Keywords:** Human behaviour, Animal behaviour

## Abstract

Inter-specific emotion recognition is especially adaptive when species spend a long time in close association, like dogs and humans. Here, we comprehensively studied the human ability to recognize facial expressions associated with dog emotions (hereafter, emotions). Participants were presented with pictures of dogs, humans and chimpanzees, showing angry, fearful, happy, neutral and sad emotions, and had to assess which emotion was shown, and the context in which the picture had been taken. Participants were recruited among children and adults with different levels of general experience with dogs, resulting from different personal (i.e. dog ownership) and cultural experiences (i.e. growing up or being exposed to a cultural milieu in which dogs are highly valued and integrated in human lives). Our results showed that some dog emotions such as anger and happiness are recognized from early on, independently of experience. However, the ability to recognize dog emotions is mainly acquired through experience. In adults, the probability of recognizing dog emotions was higher for participants grown up in a cultural milieu with a positive attitude toward dogs, which may result in different passive exposure, interest or inclination toward this species.

## Introduction

The physiological foundations of basic emotions are shared by humans and other mammals^1–5^. Emotions are often expressed through behavioural and somatic displays^4^, which serve as signals for other individuals and may have a crucial communicatory and social function in several species^6–9^. Through the expression of emotions, for instance, individuals may communicate their intentions and motivations^7^, facilitating conspecifics’ responses and the establishment and maintenance of long-term relationships^6^.

Recognizing others’ facial expressions of emotions, therefore, clearly provides fitness benefits^6,7^. For instance, an animal may become alert when another individual displays fear, as a predator or aggressive conspecifics may be nearby. Recognizing others’ emotions may also be advantageous in inter-specific interactions, such as mutualism or predator-prey interactions^10^. However, inter-specific emotion recognition may be especially challenging, as the same emotion may be displayed differently in different taxa^10^. Therefore, it is expected to be favoured by evolution when two species spend a significant amount of time in close association with each other, and each species gains fitness benefits through recognition of the other species’ emotions.

Close association between humans and domestic dogs (*Canis familiaris*) has occurred since dogs’ domestication, at least 30 000 years ago^11,12^. Dogs show remarkable communicative skills: they may use eye gaze as a communicative act^13,14^, and decipher humans’ communicative intent^15^. They can also make use of words^16^, iconic signs^17^ and human gestures^18–20^. Moreover, dogs can use acoustic and visual cues to recognize human emotions. Dogs, for instance, take the emotional expression of their owner into account when deciding whether to approach a novel object^21^. Dogs can also recognize the emotional expressions of human faces e.g.^10,22^, and integrate bimodal sensory information to discriminate positive and negative emotions from dogs and humans^23^. Importantly, dogs’ ability to recognize human emotions appears to exceed the ability of other taxa, including wolves and chimpanzees, and it may be the result of the domestication process having selected for dogs that most proficiently communicate with humans^24–26^.

In contrast, the human ability to recognize dog emotions has received only limited attention. Studies using auditory input demonstrate that humans can recognize some dog emotions, like aggressive barks to strangers^27–30^. While dogs may display their emotions through auditory signalling^31^, they also use a large range of body and facial signals, which are a primary channel for emotional transmission in several species e.g.^6,32–34^. However, several studies suggest that children and adults do not reliably understand the body signals of dogs^35–38^, and that children often mistake angry dog facial displays for happy ones^39^. Indeed, not all emotions may be equally easy to recognize. Overall, people are generally more successful at recognizing positive dog emotions, like happiness^38,40–42^, while often confusing negative emotions, like fear^38,40,41^; but see^42^. More contrasting results have emerged on the human ability to recognize dogs’ aggressive behaviour from visual cues, with positive^38,40^ and negative evidence^42^.

Furthermore, it is not clear whether previous experience with dogs is necessary for humans to recognize dog emotions^10^. According to the co-domestication hypothesis, human ability to recognize dog emotions (or at least some especially relevant ones, like angry emotions) may be supported by specially adapted mechanisms. In particular, convergent evolution would have led humans and dogs to evolve emotional displays and cognitive skills that favour reciprocal understanding and inter-specific communication, with humans selecting dogs based on their working abilities and communication skills, and humans evolving an ability to read dog emotions^13,20,43–46^. Therefore, even though direct experience with dogs (e.g. dog ownership) may still increase the ability to recognize dog emotions, this ability should be partially present also in the absence of experience. Also in this respect, experimental evidence provides contrasting results. In some studies, inexperienced humans (i.e. non-owners) were better than humans with dog experience (e.g. dog owners) at reading dog emotions^38^, reliably recognizing positive (i.e. curiosity and play) and negative (i.e. fear and social isolation) emotions^34,47^. In other studies, the ability to recognize dog emotions did not differ between dog-owners and non-owners^28,29,42^, although in some cases, and for some emotions, it did increase slightly with age and experience^30,34,41,47^; see^25^.

Here, we conducted the first comprehensive study of the human ability to recognize the facial expressions associated with dog emotions (hereafter, emotions). Firstly, we thoroughly assessed the effect of experience with dogs on the ability to recognize their emotions. “Experience with dogs” is a general concept that may encompass a variety of relationships with dogs, such as (i) ownership of a dog, (ii) growing up in or (iii) being exposed to a cultural milieu with a dog-positive attitude. By a cultural milieu with a dog-positive attitude we refer to a society in which dogs are highly integrated in human lives, and in which there is a general positive attitude toward them. In Europe, for example, dogs are generally seen as part of the family, are walked on a lead, enter homes and spend substantial amount of time with people. In contrast, in traditional communities in Muslim countries, dogs are often viewed as impure and rarely integrated as part of the family see^48,49^. Clearly, this has nothing to do with the mistaken notion that Muslims would hate dogs, and simply implies that different societies may importantly differ in their general attitude to dogs. Therefore, our study included (i) non-Muslim European dog-owners (i.e. owners who grew up in and were exposed to a cultural milieu with a dog-positive attitude), (ii) non-Muslim European non-owners (i.e. non-owners who grew up in and were exposed to a cultural milieu with a dog-positive attitude), (iii) Muslim non-owners from countries in which Islam is the majority religion, but living in Europe for at least 3 years (i.e. non-owners who grew up in a cultural milieu with no dog-positive attitude, but who were extensively exposed to one with a dog-positive attitude), and (iv) Muslim non-owners living in Morocco (i.e. non-owners who grew up in and were exposed to a cultural milieu with no dog-positive attitude). If the co-domestication hypothesis is to be supported, high performance would be expected also in groups who grew up in a cultural milieu with no dog-positive attitude, and/or were not exposed to a cultural milieu with a dog-positive attitude. However, experience with dogs may nonetheless increase human ability to recognize dog emotions. Therefore, being culturally exposed to a dog-positive attitude, growing up in such a cultural milieu and owning a dog should have an increasingly stronger positive effect on the ability to read dog emotions.

Secondly, we compared participants’ ability to recognize dog, chimpanzee and human emotions, to assess whether participants’ ability to read human emotions is more similar to their ability to read dog emotions (as predicted by the co-domestication hypothesis) or chimpanzee emotions (if emotions are recognized on the basis of shared phylogenetic history). Moreover, by testing whether chimpanzee emotions were recognized by all participants in a similar way, independently of their general dog experience, we could rule out that potential differences in the ability to recognize dog emotions were simply reflecting more general differences in participants’ overall ability to read animal emotions.

Thirdly, we assessed the effect of age on emotion recognition, by testing both children and adults. If the ability to recognize dog emotions has been selected through evolution (in line with the co-domestication hypothesis), performance in children should be similar to performance in adults, and similar in all participant groups.

## Methods

### Participants

As adult participants, we recruited 24 non-Muslim European dog-owners; 24 non-Muslim Europeans who did not own a dog and did not live in close contact with one (hereafter, non-owners); 18 Muslim non-owners from a country in which Islam is the majority religion, but that had been living in Europe for more than 3 years; and 23 Muslim non-owners living in Morocco. Through formal educational establishments and local kindergartens, we further recruited 5- and 6-year-old children. In particular, we included 23 non-Muslim European dog-owners; 31 non-Muslim European non-owners; and 23 Muslim non-owners living in Morocco. Participants belonged to both sexes, and differed in terms of their individual attitude to dogs (i.e. how much they liked dogs and considered them to be important for humans). All Muslim participants were practicing Muslims, except for one. Both in adults and children, non-Muslim European dog-owners (hereafter, EGO, with E standing for Extensive exposure to a dog-positive cultural milieu, G standing for having Grown-up in such a cultural milieu, and O standing for dog-Ownership) liked dogs the most and found them most important, followed by non-Muslim European non-owners (EGo, with o standing for no dog-ownership), Muslim non-owners in Europe (Ego, with g standing for not having grown-up in a dog-positive cultural milieu) and Muslim non-owners in Morocco (ego, with e standing for not having been exposed to a dog-positive cultural milieu; see Supplementary Material for more details).

### Materials

Stimuli consisted of frontal facial photographs of 20 different dogs, 20 different chimpanzees, and 20 different humans, on a white background. Dog pictures were taken in a park in Leipzig, Germany, noting the context in which the pictures were taken (see below), or pre-selected from real-life images from the internet, ascertaining the context by contacting the owners of the pictures. All dogs had a wolf-like face (i.e. German Shepherd, Husky and similar), with upright ears and relatively short hair. This is because other breeds may have a reduced social signaling capacity, due to brachycephaly, floppy ears or long fur in the face. Chimpanzee pictures were real-life images taken at the Wolfgang Koehler Primate Research Center in Leipzig, Germany, also noting the context in which the pictures had been taken. In this way we defined all animal emotions depending on the objective context in which the pictures had been taken, anchoring the pictures to behaviourally defined situations^38,50^. Finally, human pictures were instructed images downloaded from the AR Face Database^51^.

All the pictures were selected by the first and last authors if they both considered them typical for the contexts listed below. “Typical” referred to the fact that these facial expressions were regularly displayed in these contexts (e.g. in chimpanzees, play face during playful interactions with conspecifics; see e.g. Parr *et al*. 2006). Pictures were also sent to other seven expert colleagues, who classified them into the five different categories described below. Researchers’ agreement with the authors’ choice was very good (*r*_*s*_ = 0.91; *N* = 320, *p* < 0.001).

In line with previous literature e.g.^28–30^, we included the following 5 different emotions (displayed by 4 different individuals per species): (a) happy/playful, (b) sad/distressed, (c) angry, (d) fearful and (e) neutral. For dogs and chimpanzees, pictures had been respectively taken in the following contexts: (a) the individual was together with a trusted conspecific partner, playing with him/her; (b) the individual had been abandoned or was observing a stressful/undesirable event, like a fight; (c) the individual was in a state of excitement directly before/while attacking a conspecific; (d) the individual was afraid of being directly attacked by some stronger conspecific partner; (e) none of the previously described situations had been happening/happened in the last/next 3 minutes.

### Procedures

Research was approved by the University of Lincoln Psychology Research Ethics Committee (soprec@lincoln.ac.uk) and by the University of Jena, and the methods were carried out in accordance with the relevant guidelines and regulations. Informed consent was directly signed by adult participants. In case of children, informed consent was obtained from their parents and/or legal guardians through the kindergartens. All adults were tested with the following procedure. Before being tested, participants provided biographic information, were asked to state whether they owned a dog or had had close contact with dogs during their lives, and provided their opinion on dogs on a scale from 1 to 5 (i.e. “how much do you like dogs”, and “how important are dogs for humans”). In contrast, none of the participants had ever owned or had had extensive experience with chimpanzees (e.g. working with them, or having friends who owned a chimpanzee). After that, the Experimenter (E) instructed participants in the procedure for the two tasks.

In the first task, we adapted the procedure from Pongracz and colleagues^29^. E sat in front of the participants and provided them with a pen and coding sheets with which to note their answers. For each participant E had a set of 30 pictures (see Supplementary Material for more information). Each one was shown to the subject for up to 30 s, or until the subject rated the picture on the coding sheet, on a 1–5 scale see e.g.^29^, for each of the 4 emotions above: happiness, sadness, anger, fear. In the second task E repeated the procedure, showing participants the same set of 30 pictures, in the same order. However, participants had to specify in which of the 5 contexts listed above the picture had been taken. Adults’ responses in both tasks were analysed together in the same model (see below). To make it more age appropriate see^40,52^, children were tested with a simplified version of the first task, in which they were shown 15 pictures and had to attribute each picture to one of the five emotions (happiness, sadness, anger, fear, and neutral), as read aloud by E.

### Statistics

Analyses were conducted using generalized linear mixed models^53^ with the lme4 package in R software (version 3.2.3)^54^. Continuous variables were *z*-transformed to facilitate model convergence. We used a likelihood ratio test^55^ to compare full models with null models. When full models differed significantly from null models, likelihood ratio tests were conducted to obtain the *p* values for each test predictor via single-term deletion^56^. Post-hoc comparisons were then conducted using Tukey tests (below, only significant post-hoc tests are reported). No convergence issues were detected. To rule out collinearity, we used variance inflation factors (VIF^57^), which were good (maximum VIFs across all models = 1.85). No random slopes were included to avoid convergence issues.

Model 1 investigated how emotion recognition by children is affected by their general experience with dogs, depending on the species and emotion observed. Given that the dependent variable was binary (i.e. 0 for an incorrect choice and 1 for a correct choice), models were run with a binomial structure. General experience (i.e. EGO, EGo, ego) emotion (anger, fear, happiness, neutrality, sadness), species (dogs, chimpanzees, humans), and their 2- and 3-way interactions were test predictors. As control predictors we entered participants’ gender (male or female), number of trials (1 to 15) and the proportion of trials in which each participant selected that specific emotion in the species (to control for the fact that participants who answer with a certain emotion in most trials will also have a higher probability to correctly recognize that emotion, despite having no greater sensitivity to that emotional expression). We further included child ID as a random effect, to account for the non-independence of data points.

Model 2 investigated how emotion recognition by adults is affected by their general experience with dogs, depending on the species and emotion observed. We used the same binary dependent variable (i.e. individual response at recognizing emotions) and test predictors as in Model 1 (i.e. general experience with dogs, emotion, species and their 2- and 3-way interactions), further including task type (i.e. Task 1 or 2) among the test predictors (as adults were administered two tasks), and a fourth factor level for experience (i.e. Ego). We also included the same control predictors as fixed effects (i.e. proportion of trials in which each participant selected that specific emotion in the species, participants’ gender and number of trials, from 1 to 30), further including subject’s age (as this varied across adult participants; see Supplementary Material). Furthermore, we included adult ID as a random effect.

If three-way interactions were not significant, they were downgraded to the two-way interactions experience x species and emotion x species. Post-hoc comparisons in Models 1 and 2 assessed whether participants with different general dog experience differed at recognizing specific emotions in different species. For each emotion, we also analysed whether participants with different general dog experience recognized dog emotions differently than in the other two species. Only for dog emotions, we further addressed which emotions were easier to evaluate by participant groups with different general dog experience.

Finally, to assess whether individual ability to recognize emotions is consistent across the three species (humans, chimpanzees and dogs), we calculated the average of correct responses for each subject and species and used Spearman exact tests, separately for adults and children.

## Results

### Model 1 – Children

The comparison between the full and null model was significant (GLMM: *χ*^2^ = 209.76, df = 44, *p* < 0.001), but not the three-way interaction experience*emotion*species (GLMM: *χ*^2^ = 19.35, df = 16, *p* = 0.251). After downgrading the interaction, the two-way interaction emotion x species was significant (GLMM: *χ*^2^ = 68.36, df = 8, *p* < 0.001), but not the interaction experience x species (GLMM: *χ*^2^ = 6.95, df = 4, *p* = 0.139; see Table 1).

**Table 1:** Results of Models 1 and 2, for children and adults (respectively), with emotion recognition as the dependent variable.

Test category	Children			Adults		
	*χ* ^2^	df	*P*	*χ* ^2^	df	*P*
Experience * Species	6.95	4	0.139	34.79	6	<0.001*
Emotion * Species	68.36	8	<0.001*	165.69	8	<0.001*
Task type	—	—	—	8.65	1	0.003*
Trial number	7.32	1	0.007*	11.65	1	<0.001*
Proportion of trials in which the emotion was selected	453.87	1	<0.001*	1321.32	1	<0.001*
Subject’s gender	0.31	1	0.576	2.08	1	0.149
Subject’s age	—	—	—	0.00	1	0.975

Significant results are marked with. *Significant test predictors are also in bold.

**Figure 1:**
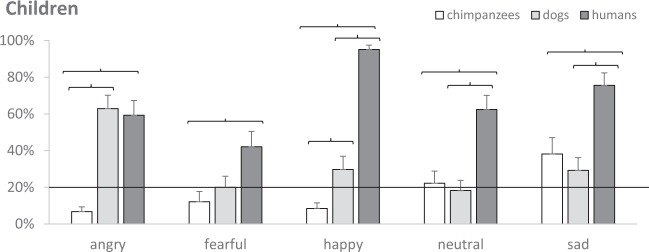
For each species (dogs, humans, chimpanzees), mean estimated probability (+SE) of children recognizing different emotions (angry, fearful, happy, neutral, sad). Parentheses indicate significant post-hoc comparisons, and the continuous line chance level.

Post-hoc analyses see^58^ were run to assess whether emotional expressions were more easily recognized in certain species, across all participants (Fig. 1). All five emotions were recognized more easily in humans than in chimpanzees (all *p* < 0.004). Neutral and sad emotions were recognized more easily in humans than in dogs (both *p* < 0.001). Happy emotions were recognized more easily in humans than in the other species, but also in dogs more than chimpanzees (both *p* ≤ 0.005). Finally, angry emotions were recognized in dogs like in humans, and more than chimpanzees (*p* < 0.001).

### Model 2 – Adults

The comparison between the full and null model was significant (GLMM: *χ*^2^ = 1045, df = 60, *p* < 0.001) but not the three-way interaction experience*emotion*species (GLMM: *χ*^2^ = 30.59, df = 24, *p* = 0.166). After downgrading the interaction, both the two-way interaction emotion x species (GLMM: *χ*^2^ = 165.69, df = 8, *p* < 0.001) and the interaction experience x species (GLMM: *χ*^2^ = 34.79, df = 6, *p* < 0.001) were significant. Also the task type was significant (GLMM: *χ*^2^ = 8.65, df = 1, *p* = 0.003), indicating that recognizing the context in which the picture was taken was significantly easier than directly naming emotions (see Table 1).

**Figure 2:**
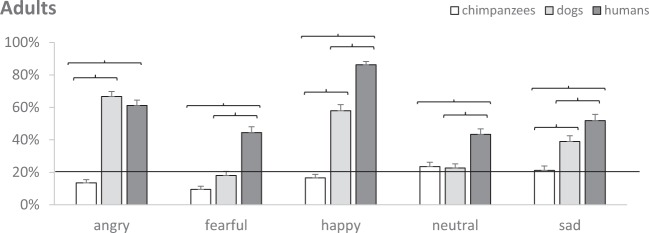
For each species (dogs, humans, chimpanzees), mean estimated probability (+SE) of adults recognizing different emotions (angry, fearful, happy, neutral, sad). Parentheses indicate significant post-hoc comparisons, and the continuous line chance level.

Post-hoc analyses see^58^ were run to assess whether experience with dogs affected adults’ ability to recognize emotions in certain species (Fig. 2). Non-Muslims (EGO, EGo) were better than Muslims (Ego, ego) at recognizing dog emotions (all *p* < 0.001). Non-Muslims (EGO, EGo) were also better than Muslims (Ego, ego) at recognizing human emotions, which were displayed by Caucasian actors (all *p* < 0.001). Experience with dogs had no effect on the ability to recognize emotions in chimpanzees.

**Figure 3:**
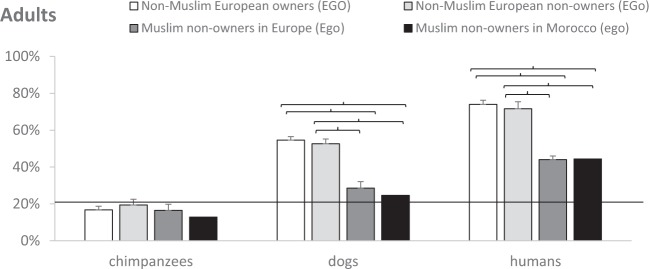
For each experience group (i.e. non-Muslim European owners EGO, non-Muslim European non-owners EGo, Muslim non-owners in Europe Ego, Muslim non-owners in Morocco ego), mean estimated probability (+SE) of adults recognizing emotions in different species (chimpanzees, dogs and humans). Parentheses indicate significant post-hoc comparisons, and the continuous line chance level.

Post-hoc analyses were further run to assess whether emotional expressions were more easily recognized in certain species by all participants (Fig. 3). As in children, all five emotions were recognized more easily in humans than in chimpanzees (all *p* < 0.001). Neutral and fearful emotions were recognized more easily in humans than in dogs (both *p* < 0.001). Happy and sad emotions were recognized more easily in humans than in the other species, but also in dogs more than chimpanzees (both *p* ≤ 0.023). Finally, like in children, angry emotions were recognized in dogs like in humans, and more than chimpanzees (*p* < 0.001).

### Spearman exact tests

Finally, we assessed whether individual ability to recognize emotions was consistent across the three species. For adults, the individual average of correct responses correlated between species of stimuli (dogs-chimpanzees: *ρ*_*s*_ = 0.323, *n* = 89, *p* = 0.002; dogs-humans: *ρ*_*s*_ = 0.644, *n* = 89, *p* < 0.001). In contrast, children’s average of correct responses did not correlate between species of stimuli (dogs-chimpanzees: *ρ*_*s*_ = 0.002, *n* = 77, *p* = 0.987; dogs-humans: *ρ*_*s*_ = 0.189, *n* = 77, *p* = 0.098).

## Discussion

Our results indicate that the ability to recognize dog emotions is mainly acquired through experience. In particular, children’s ability to recognize dog emotions was similar across all participants, with no effect of general experience with dogs. Children had more trouble recognizing dog emotions than human emotions (except for anger), and were equally bad at recognizing dog and chimpanzee emotions (except for angry and happy emotions, which were recognized in dogs better than in chimpanzees). Except for anger and perhaps happiness, therefore, children appear able to only limitedly recognize dog emotions, regardless of their general experience with dogs. In adults, in contrast, the ability to recognize emotions strongly varied depending on their general experience with dogs, but also on the emotion and the species of the stimuli observed. In particular, participants with more general dog experience (i.e. EGO and EGo, who grew up in and were exposed to a cultural milieu with a dog-positive attitude, regardless of whether they owned a dog) were overall more proficient at recognizing dog emotions than participants with less general dog experience (i.e. Ego and ego, who grew up in a cultural milieu with no dog-positive attitude). These differences did not hold when assessing chimpanzee emotions. Finally, as in children, adults recognized all dog emotions worse than human emotions (except for anger), but angry, sad and happy emotions were recognized better than in chimpanzees, suggesting a possible increase through age in the ability to recognize dog emotions.

All children recognized dog emotions in the same way, independently of their experience with dogs. Moreover, except for anger and happiness, children had just as much trouble recognizing dog emotions as chimpanzee emotions. These results suggest that the ability to read dog emotions does not manifest spontaneously in young children (but see below for further discussion), and it is mainly acquired over the course of development. In adults, in contrast, general experience with dogs had a clear positive effect on the ability to understand dog emotions. Participants growing up in a cultural milieu with a dog-positive attitude (EGO and EGo) were overall more proficient at recognizing dog emotions than other participants (Ego and ego). These results are noteworthy because they suggest that it is not necessarily direct experience with dogs (i.e. dog ownership) that affects humans’ ability to recognize their emotions e.g.^28,29,38,42,59^, but rather the cultural milieu in which humans develop. Growing up in a cultural milieu in which dogs are viewed as highly important for humans, and are highly integrated in human lives, may result in different passive exposure, or different interest and inclination toward this species. Therefore, possible cultural differences in the ability to read dog emotions only emerge through development (at least after 6 years of age), when the effects of growing up in a cultural milieu with a different attitude toward dogs start affecting human ability to recognize their emotions. Crucially, all adult participants recognized chimpanzee emotions in a very similar way, independently of their general dog experience, suggesting that our results were not simply reflecting more general differences in participants’ overall ability to read animal emotions.

An important exception to this pattern are anger and happiness, which were reliably recognized also by children, regardless of their previous general experience with dogs. These results seem to support the co-domestication hypothesis, in that even children with minimal experience (i.e. young age, no direct experience with dogs, no cultural milieu with a dog-positive attitude) correctly interpret some dog emotions. The ability to recognize anger is clearly adaptive, as it provides immediate fitness benefits (i.e. reduced risk of receiving aggression) by conveying crucial information about possibly dangerous situations, and thus bears higher survival costs^27,30,38^. However, it is also possible that our results simply reflect the fact that humans quickly learn to recognize anger through experience. The fact that even young Muslim children in Morocco could successfully recognize dog anger provides more support to the hypothesis of this ability being supported by specially adapted mechanisms that operate largely independent of specific experiences. However, future studies should provide stronger evidence in this sense, by for instance testing even younger children.

Also for adults, not all dog emotions were as easy to recognize. Adult participants were generally proficient at recognizing happy emotions, but not fearful ones. These results are in line with previous studies, also suggesting that dog fearful emotions may be especially hard to read e.g.^30,40,41^, while happy/friendly emotions are easier to decode^25,40,41^. Adults were also generally proficient at recognizing angry emotions, like children. In children, angry and happy emotions were recognized more easily than sad and fearful ones. These results seem to expand a pattern, according to which humans recognize happiness and anger in other humans relatively early in life, while fear recognition follows a much slower developmental trajectory^60^.

Overall, children’s performance only marginally matched adults’ performance, as only adults highly varied in their response depending on general experience, species and emotion. This suggests that, although some emotions are recognized early on independently of previous experience, learning is crucial to improve dog emotion recognition. These results confirm previous studies, showing that younger children are less proficient at reading dog emotions e.g.^40^. If the ability to recognize emotions is acquired through development, it is therefore no surprise that culture may play a major role in the skills acquired, as not all emotions may be culturally relevant e.g.^61^.

Finally, our results raise four additional considerations. Firstly, inter-individual differences in emotion recognition were maintained across species, but only in adults, with participants that were better able to recognize emotions in one species also being better able to recognize them in the other ones. This is in line with other findings showing that humans perceive human and dog facial expressions in a similar way e.g.^25^. Secondly, success at recognizing emotions differed depending on the task administered, with adults performing better overall when asked to recognize the context in which the pictures were taken, as compared to when asked to name the emotions observed see e.g.^62^. This suggests that tasks using context cues may be more efficient in catching human ability to read emotions. Thirdly, emotions were generally recognized better in humans than in dogs by all participants, but Muslim participants performed worse than non-Muslims at recognizing human emotions. This was likely due to a limitation of our study, in that the human stimuli we used only depicted white Western models see^63–67^. Fourthly, all participants were rather bad at recognizing chimpanzee emotions. Although this may seem unexpected, it is important to note that chimpanzees and humans, despite being closely related and thus sharing physical and functional similarities^68–71^, have facial emotions that differ in substantial ways (e.g. ear and head movements play a larger role in chimpanzee facial expressions^71,72^).

Unfortunately, our study also presented several limitations. Firstly, pictures of emotional expressions were selected based on the authors’ and other expert colleagues’ rating. Although the selection was based on objective criteria (see above), future studies may benefit from using different approaches for the stimuli selection, like FACS e.g.^68^. Secondly, all dogs included in our stimuli had a German shepherd-like face, because morphological features of these breeds (e.g. hair, ears) ensure that emotional expressions are well visible. However, future studies should include a larger variety of dog breeds, and specifically test whether experience with specific dog breeds easily transfers to the ability of recognizing emotions in different breeds. Thirdly, future studies should also include participants with a broader range of experience with dogs, also including participants not owning a dog, but with extensive experience with dogs (e.g. experts working with dogs, non-experts living in close contact to dogs), and possibly participants from other cultural milieus which may show more diverse attitudes to dogs. Fourthly, the stimuli we used depicted different individual dogs for the different expressions, creating a potential confound between the emotional expressions and the identity of the dog. As participants across all tested groups (EGO, EGo, Ego, ego) were exposed to exactly the same stimuli, this limitation cannot explain the results obtained. However, future studies should ideally present participants with pictures of the same individuals showing the different emotional expressions. Finally, before being tested, participants were asked about their experience and relationship with dogs. Although this approach was necessary to select participants for the different tested groups (EGO, EGo, Ego, ego), we cannot rule out that these questions might have affected participants’ response in the tasks. Therefore, future studies should find a different approach to recruit participants.

In conclusion, our study provides partial support for the co-domestication hypothesis, but also shows that the ability to recognize dog emotions is largely acquired through general experience with dogs. More than direct experience with dogs (i.e. ownership), the cultural milieu in which participants grew up likely determined the interest with which humans attended to dogs and were therefore able to pick up subtle cues that facilitated emotional recognition. Future studies should further investigate exactly which cultural aspects affect this ability. Moreover, it will be important to use different procedures to confirm these results, by for instance comparing performance with both instructed and real-life stimuli, and both facial and body expressions e.g.^30,40^; see^73^. This will not only allow us to better understand inter-cultural variation in emotion recognition, but will also provide us with useful hints to facilitate inter-specific communication and reduce the occurrence of harmful or negative incidents between humans and dogs caused by humans’ inability to read dog signals e.g.^74^.

## Supplementary information

Supplementary Information
