# Supplementary material for: The ability to recognize dog emotions depends on the cultural milieu in which we grow up
Source: Sci Rep. 2019 Nov 11;9:16414. doi: 10.1038/s41598-019-52938-4 (PMC6848084; doi:10.1038/s41598-019-52938-4)
Supplement: Supplementary file 1 — Supplementary Information [file 41598_2019_52938_MOESM1_ESM.pdf]

Title: The ability to recognize dog emotions depends on the cultural milieu in which we grow up.

Federica Amici [1,2,3,*], James Waterman [4], Christina Maria Kellermann [3,5], Karimullah Karimullah [2], Juliane Bräuer [6,7]

[1] Research Group "Primate Behavioural Ecology", Department of Human Behavior, Ecology and Culture, Max Planck Institute for Evolutionary Anthropology, Leipzig, Germany

[2] Behavioral Ecology Research Group, Institute of Biology, Faculty of Life Science, University of Leipzig, Leipzig, Germany

[3] Leipzig Research Center for Early Child Development, University of Leipzig, Leipzig, Germany

[4] School of Psychology, University of Lincoln, Lincoln, UK

[5] Faculty of Social and Behavioral Sciences, Friedrich Schiller University, Jena, Germany

[6] Department of Linguistic and Cultural Evolution, Max Planck Institute for the Science of Human History, Jena, Germany

[7] Friedrich Schiller University, Department of General Psychology and Cognitive Neuroscience, Jena, Germany

METHODS

Participants. We recruited 24 non-Muslim British adults who owned a dog (mean age: 21 years and 11 months), 24 non-Muslim British adults who did not own a dog and did not live in close contact with one (mean age: 20 years and 6 months), 18 Muslim adults from a Muslim country but that had been living in Germany for more than 3 years (mean age: 29 years and 11 months), and 23 Muslim adults living in Morocco, who did not own a dog and did not live in close contact with one (mean age: 35 years and 9 months). We further recruited 23 non-Muslim German children who owned a dog (mean age: 5 years and 6 months), 31 non-Muslim German children who did not own a dog or live in close contact with one (mean age: 5 years and 8 months), and 23 Muslim children living in Morocco, who did not own a dog and did not live in close contact with one (mean age: 5 years and 7 months).

Non-Muslim adults were recruited through a database from the Lincoln University (UK), while non-Muslim children were recruited thanks to the children data-base of the Leipzig Research Center for Early Child Development (Germany). Muslim adults in Germany and Morocco were opportunistically recruited by the authors thanks to the contacts of the fourth and the second author, respectively. Finally, Muslim children in Morocco were recruited through formal educational establishments and local kindergartens by the first author.

Participants differed in terms of their individual attitude to dogs (i.e. how much they liked dogs and considered them to be important for humans; both assessed on a scale from 1 to 5). In adults, non-Muslim European owners liked dogs the most (4.4), followed by non-Muslim European non-owners (3.2), Muslim non-owners in Europe (2.5) and Muslim non-owners in Morocco (2.1). In children, non-Muslim European owners liked dogs the most (4.8), followed by non-Muslim European non-owners and Muslim non-owners in Morocco (3.3). The same pattern

was found for participants' assessment of dogs' importance. In adults, non-Muslim European owners found dogs to be very important (4.1), followed by non-Muslim European non-owners (3.5), and Muslim non-owners in Europe and in Morocco (2.6). In children, non-Muslim European owners found dogs to be very important (4.5), followed by non-Muslim European non-owners (3.8) and Muslim non-owners in Morocco (2.7). This pattern perfectly mirrors the idea that non-Muslim European dog owners, non-Muslim European non-owners, Muslim non-owners in Europe and Muslim non-owners in Morocco have a different attitude toward dogs, which appears to be mostly positive in non-Muslim European dog owners and gradually decreases in the other participant groups.

Materials. Pictures of chimpanzee and dog emotions were selected by several experts in dog and ape cognition, for being "typical" in the specific emotional context. Photographs were transformed through Photoshop to exclude the background and the body, leaving only the face well visible on a white background, and they were finally printed and laminated.

Dog pictures were taken in a park in Leipzig, Germany, noting the context in which the pictures were taken (see below) and obtaining written consent from the dog owners. Other dog pictures were pre-selected from real-life images from the internet; after pre-selection, we contacted the owners of the pics to ascertain the context in which the picture had been taken and to obtain their written consent to use the pictures for the purpose of this study. In all pictures, depicted dogs belonged to different breeds, but were always characterized by a German shepherd-like facial appearance, with short hair in the face and pricked ears.

Procedures. Research was approved by the University of Lincoln Psychology Research Ethics Committee (soprec@lincoln.ac.uk), by the LFE and by the University of Jena, and is in line with British Psychological Association (BPS) guidelines. Adult participants were tested

individually in a quiet room (at the University, for non-Muslim British participants; at home and in their offices, for Muslim participants in Europe; and in a local hotel, for Muslim participants in Morocco). Children were also tested in a quiet room in their kindergartens. All participants were tested in their native language by one experimenter, with the help of a local translator in Morocco. Before data collection participants were informed that their data would be anonymously used and published for a study on dog emotion recognition, that participation was on a completely voluntary basis, and that at any time during the testing period they could interrupt participation and ask for the deletion of all the collected data. Written consent was directly signed by adult participants. In case of children, parental consent was obtained prior to the test through the kindergartens. Participants were always free to leave the task if they no longer wanted to engage with the experimenter, although this never happened. All data have been anonymously stored and are available on request.

In the first task for adults, the experimenter had a series of 30 pictures for each participant. The order of the pictures was pseudo-randomised across participants, but the same emotion and/or species was never presented more than three times in a row. We opted to show participants only half of the pictures (i.e. 30 instead of 60), to avoid too long testing sessions and maintain motivation high during the tasks. Crucially, the 60 pictures had been previously organized in 6 different orders, so that, across these possible orders, all 60 pictures were shown the same number of times. The same number of participants was then tested with these 6 orders, both within and across tested groups, ensuring that exposure to the different pictures was identical across testing groups.

The experimenter sat in front of the participants, and provided them with a pen and coding sheets, with which they had to note their answers. In the coding sheets, definitions of emotions

were always visible. The experimenter showed the first picture of the series to the subject by holding it vertically in front of him/her for up to 30 seconds. During these 30 seconds the subject was required to rate the picture on a 5-point scale (not at all, a little, some, much, very much), for each of the 4 basic emotions above (happiness, sadness, anger, fear). When the time had elapsed, or the subject noted the answer, the picture was laid face down and after 10 seconds a new picture was presented and the procedure repeated. After a small break (3 minutes) adults were also tested in the second task.

In order to simplify the procedure and make it age appropriate [see 1], we only tested children with a simplified version of the first task. Firstly, children did not have to give a quantitative judgement of emotion, but instead had to attribute each picture to one of the five emotions (happiness, sadness, anger, fear, and neutral). Secondly, in order to keep test sessions as brief as possible, only 15 pictures were shown (i.e. one per emotion and species, with the 4 sets of always different pictures being used in a randomized and counterbalanced order across participants). Thirdly, children did not have to read the coding sheet and write the answer, as the researcher read the emotions aloud and then noted down the children's answer. The second task was not carried out with child participants, due to the abstract nature of the task; young children are able to recognise emotional expression but are less likely to successfully deal with emotional perspective taking tasks, requiring more complex verbal and perspective taking skills [2-3].

Given the impressing amount of data, we opted to analyse adult data in both tasks within a single model, adding task type as predictor. For this reason, responses in the first task, which had been assessed by participants rating them on a 1-5 scale for happiness/playfulness, sadness, anger and fear, were coded as follows: for each picture, the emotion associated to the highest score was considered correct if it corresponded to the emotion shown in the picture. If, for the same picture,

the highest score was associated to more than one emotion, the response was coded as correct if the emotion shown in the picture was neutral. In the second task, a response was considered correct if the context selected by the participant corresponded to the one in which the picture had been taken. Below, we present figures with raw data from children (Figure S1) and adults, separately for Task 1 (Figure S2) and Task 2 (Figure S3).

FIGURE S1. For each species (dogs, humans, chimpanzees), mean percentage (+ SE) of correct choices by children in Task 1, as a function of emotion (angry, fearful, happy, neutral, sad), and separately for each experience group (i.e. non-Muslim European owners, non-Muslim European non-owners, Muslim non-owners in Morocco). The continuous line represents chance level.

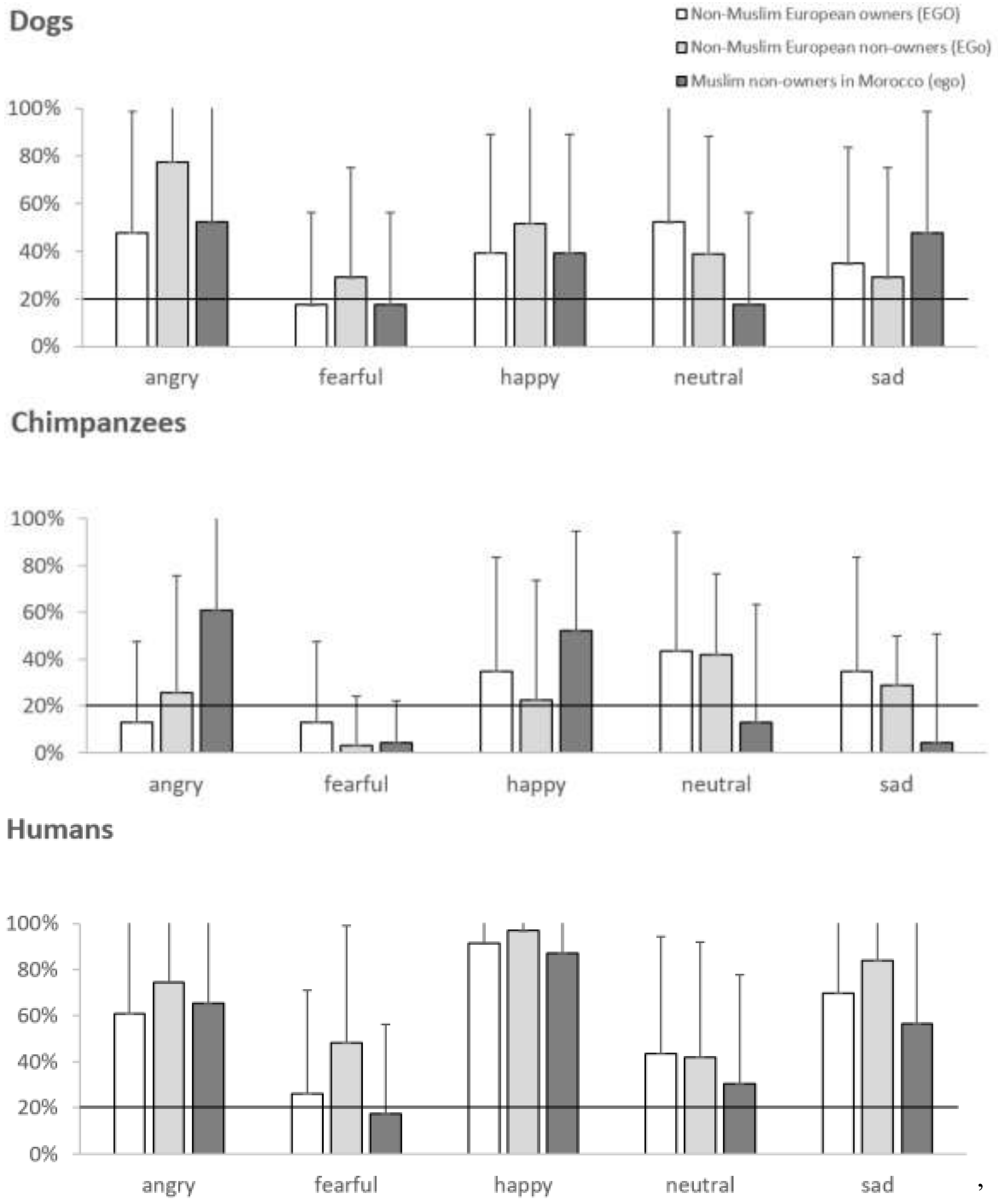

FIGURE S2. For each species (dogs, humans, chimpanzees), mean percentage (+ SE) of correct choices by adults in Task 1, as a function of emotion (angry, fearful, happy, neutral, sad), and separately for each experience group (i.e. non-Muslim European owners, non-Muslim European non-owners, Muslim non-owners in Morocco). The continuous line represents chance level.

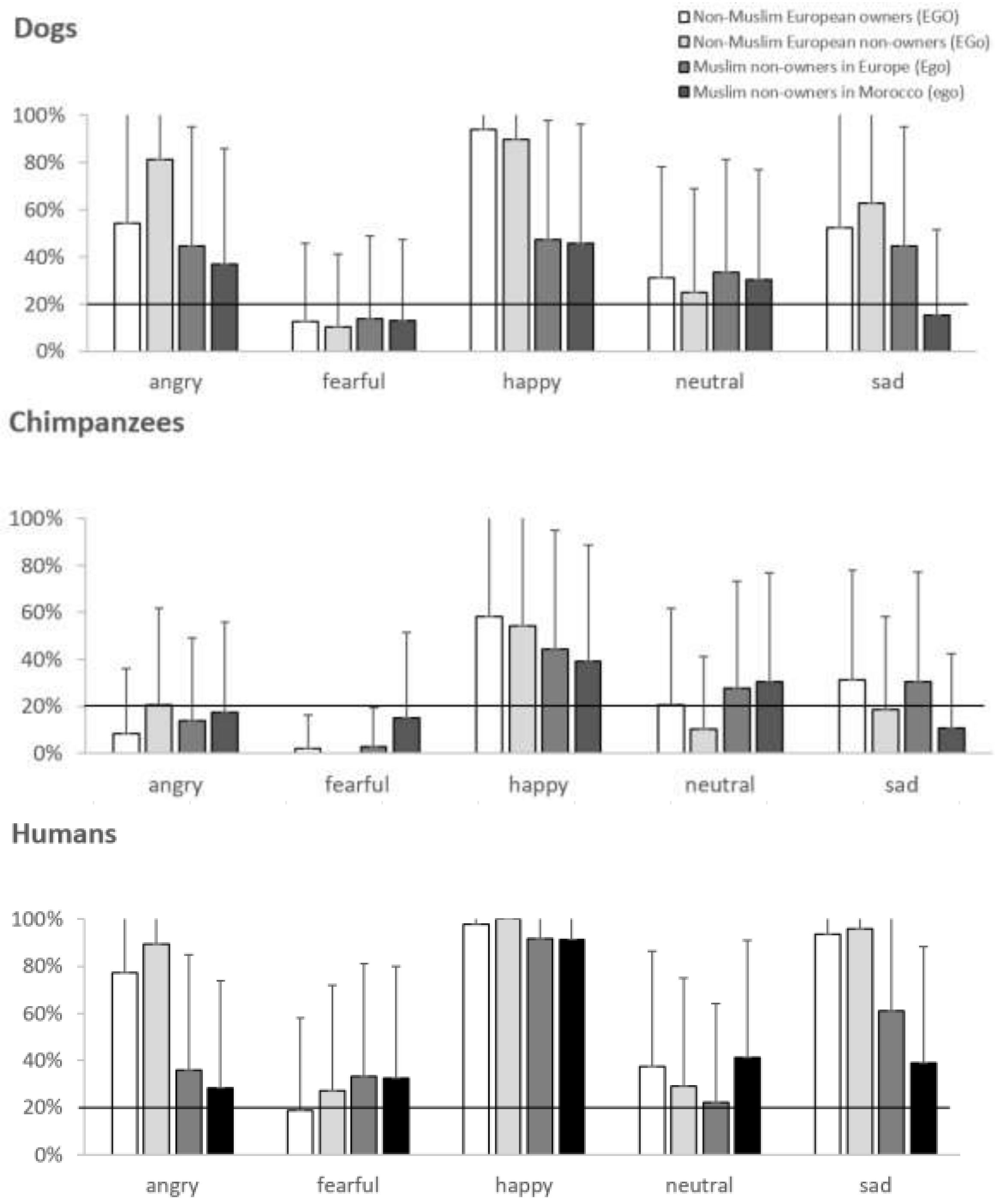

FIGURE S3. For each species (dogs, humans, chimpanzees), mean percentage (+ SE) of correct choices by adults in Task 2, as a function of emotion (angry, fearful, happy, neutral, sad), and separately for each experience group (i.e. non-Muslim European owners, non-Muslim European non-owners, Muslim non-owners in Morocco). The continuous line represents chance level.

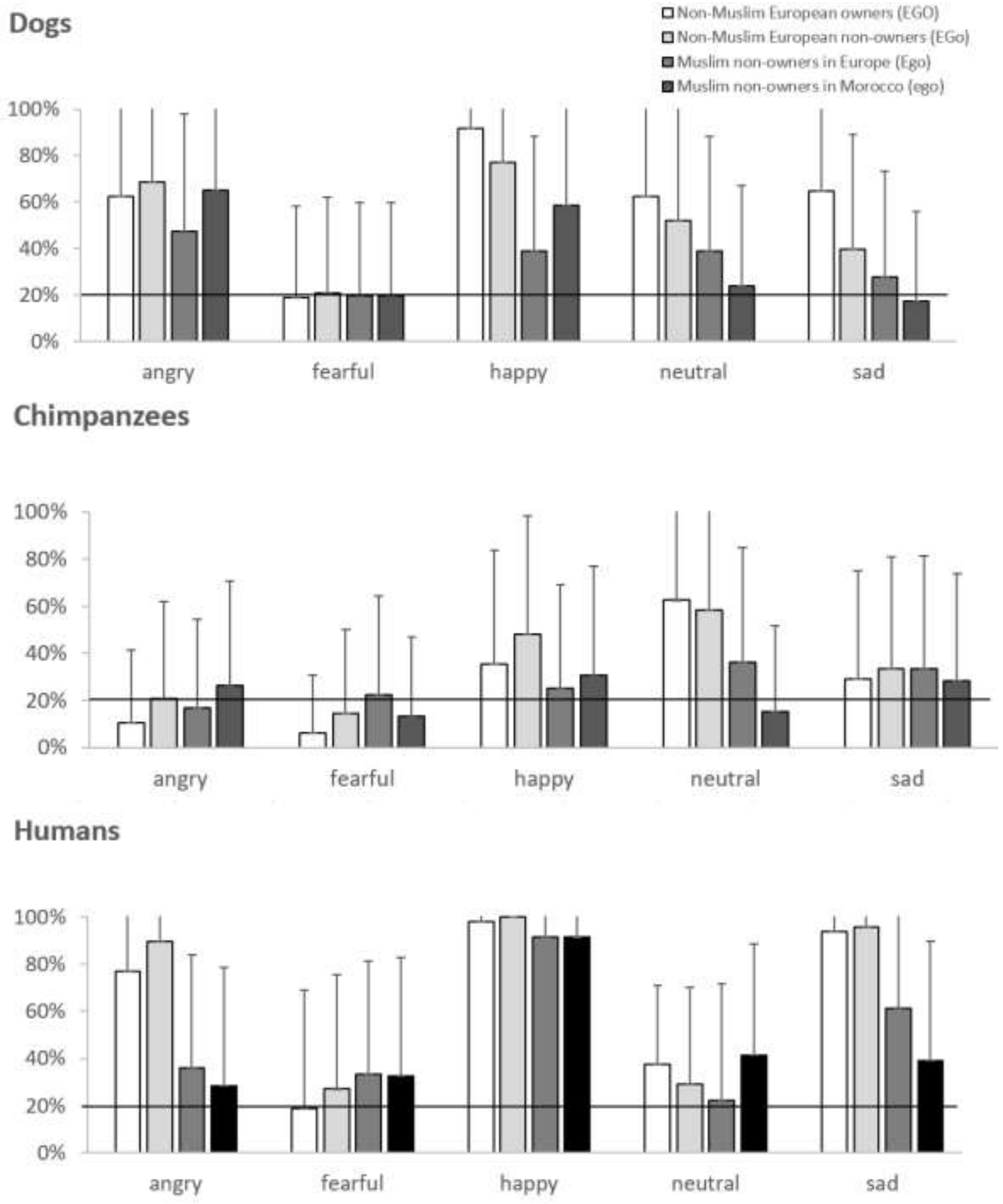

REFERENCES

1. Lakestani, N. N., Donaldson, M. L. & Waran, N. Interpretation of dog behavior by children and young adults. *Anthrozoös* **27**, 65-80 (2014).
2. Chronaki, G., Garner, M., Hadwin, J. A., Thompson, M. J. J., Chin, C. Y., Edmund, J. S. & Sonuga-Barke, E. J. S. Emotion-recognition abilities and behavior problem dimensions in preschoolers: evidence for a specific role for childhood hyperactivity. *Child Neuropsychol.* **21**, 25-40 (2015).
3. Pons, F., Harris, P. L. & de Rosnay, M. Emotion comprehension between 3 and 11 years developmental periods and hierarchical organization. *Eur. J. Dev. Psychol.* **1**, 127-152 (2004).
